# Pancreatin-Cetyl Pyridinium Chloride Digestion and Decontamination Method; A Novel, Sensitive, Cost-Effective Method for Culturing *Mycobacterium tuberculosis*

**DOI:** 10.3390/microorganisms9102025

**Published:** 2021-09-24

**Authors:** Pottathil Shinu, Anroop B. Nair, Snawar Hussain, Mohamed A. Morsy, Wafaa E. Soliman

**Affiliations:** 1 Department of Biomedical Sciences, College of Clinical Pharmacy, King Faisal University, Al-Ahsa 31982, Saudi Arabia; snawar76@gmail.com (S.H.); weahmed@kfu.edu.sa (W.E.S.); 2 Department of Microbiology, M.M. Institute of Medical Sciences and Research, Maharishi Markandeshwar (Deemed to Be University), Mullana-Ambala 133207, India; 3 Department of Pharmaceutical Sciences, College of Clinical Pharmacy, King Faisal University, Al-Ahsa 31982, Saudi Arabia; anair@kfu.edu.sa (A.B.N.); momorsy@kfu.edu.sa (M.A.M.); 4 Department of Pharmacology, Faculty of Medicine, Minia University, El-Minia 61511, Egypt; 5 Department of Microbiology and Immunology, Faculty of Pharmacy, Delta University for Science and Technology, Mansoura 11152, Egypt

**Keywords:** pulmonary tuberculosis, pancreatin digestion and decontamination method, non-tuberculous mycobacteria, *Mycobacterium tuberculosis*

## Abstract

The present study evaluated the performance of newly developed pancreatin-cetylpyridinium chloride (pancreatin-CPC) digestion and decontamination method (DDM) with N-acetyl L-Cysteine-sodium hydroxide (NALC-NaOH) DDM for isolation of Mycobacteria from clinically suspected pulmonary tuberculosis (PTB) patients. For the study, sputum samples (*n* = 613) obtained from clinically suspected PTB cases were subjected to direct microscopy, pretreatment with NALC-NaOH DDM (reference method), and pancreatin-CPC DDM followed by culture, and the data were analyzed. The direct microscopy illustrated diagnostic accuracies of 60.4% (sensitivity), 99.77% (specificity), 98.9% (positive predictive value) and 88.3% (negative predictive value), respectively (against culture) for the detection of Mycobacterial species. The pancreatin-CPC DDM showed competitive diagnostic accuracies (against NALC-NaOH DDM) of 99.32% (sensitivity), 94.07% (specificity), 85.05% (positive predictive value), and 99.76% (negative predictive value), respectively, for the isolation of Mycobacterial species. In conclusion, pancreatin-CPC DMM was a highly sensitive, technically simple, and cost-effective method, suggesting its competence to substitute the currently used NALC-NaOH DDM.

## 1. Introduction

Tuberculosis (TB) continues to be a notable health menace across the globe despite effective treatment strategies endorsed by the World Health Organization [1]. Further, early detection and isolation of *Mycobacterium tuberculosis* (MTB) in culture is necessary for accurate detection of tuberculosis (TB). However, isolation of MTB and other non-tuberculous mycobacteria (NTM) are hindered due to the presence of microbial flora in the patients’ samples resulting in delayed confirmation of tubercle bacilli [2,3]. Currently, N-Acetyl L Cysteine (NALC)-sodium hydroxide (NaOH) is the extensively used pre-treatment technique to isolate MTB from clinical specimens. However, the literature indicates that pre-treatment of clinical samples with NaOH may kill a significant number of tubercle bacilli along with other contaminating organisms [2,4,5].

Several pre-treatment methods have been investigated to isolate mycobacteria from sputum samples [2,4,5,6,7,8,9,10,11]. However, the complexity and low diagnostic accuracy of these methods limited their use in mycobacterial laboratories [6,9]. This endorses the significance of extensive research to develop highly sensitive and technically apparent methods for culturing MTB from clinical samples. This study investigated the performance of pancreatin- cetylpyridinium chloride (pancreatin-CPC DDM) with NALC-NaOH DDM to isolate TB bacilli from sputum samples obtained from clinically suspected pulmonary tuberculosis (PTB) patients. The NALC-NaOH DDM possesses various limitations such as being bulky, technically complex, the toxicity of NaOH against few strains of MTB, and instability of NALC solution (stable for 24 h once prepared). However, these problems can be overcome using pancreatin-CPC DDM, which is technically simple, easy to perform, nontoxicity of CPC against MTB strains, and stability of pancreatin-CPC solution (shelf life of one week once constituted. In the pancreatin- CPC DDM, pancreatin utilizes the property of mucolytics (for the lysis of sputum samples), and the other act as a disinfectant. Pancreatin, which is a mixture of various types of digestive enzymes (secreted by exocrine cells of the pancreas) and is capable of lysing carbohydrates, lipids, and proteins as well [12]. CPC, a mild disinfectant, which is specifically active against gram-positive bacteria [6]. The mucolytic property of pancreatin and disinfecting capacity of CPC is utilized in the pancreatin-CPC DDM. Therefore, this evaluation was aimed to compare the performance of two DDMs in the detection of tubercle bacilli from sputum samples. 

## 2. Materials and Methods

### 2.1. Study Setting

A total of 613 consecutive sputa [two sputa (spot and morning) samples per patient] were obtained from suspected cases of PTB patients, who were attending the Revised National Tuberculosis Control Program (RNTCP) testing center at M. M. Institute of Medical Sciences, Ambala (India). The current study included all patients, who presented with a productive cough that lasts longer than two weeks or more or the patients as instructed by the physician. However, patients who were undergoing anti-tubercular treatment (ATT) were excluded from the study. All the sputum samples used in the study were obtained for direct Acid-fast bacilli (AFB) staining at the RNTCP center. AFB smear reports were distributed to the patients after the microscopic examination. Further, all the sputum was refrigerated overnight at 4–8 °C until used. Informed consent of the patients was obtained before sputum collection. After dispatching the AFB smear report, all the sputum samples were transported to the Department of Microbiology, M. M. Institute of Medical Sciences and Research, Ambala (India) for culture.

### 2.2. Laboratory Methods

#### 2.2.1. Homogenization of Sputum Samples

Both sputum specimens (morning and spot) collected from each patient were pooled to obtain sufficient quantity (4–6 mL) and similar quality. These mixed sputum samples were homogenized for one min using a disposable Pasteur pipette followed by vortexing of a 3 min duration to assure the equal distribution of mycobacteria. All the procedures involving specimen segregation, smear preparation, and culture were carried out in a biological safety cabinet (Class II A) (Sterile Tech, Chennai, India). This homogenized sputum specimen was separated into three aliquots, namely; direct Auramine ‘O’ staining and other two aliquots used for culture by NALC-NaOH DDM and pancreatin-CPC DDM. 

#### 2.2.2. Direct Smear Microscopy

For the light-emitting diode fluorescent microscopy (LED FM), direct Auramine ‘O’ smears were prepared using a 0.5 mL homogenized sputum sample on a clean glass slide. Two independent technicians under 400× magnification (Primo Star iLED, Carl Zeiss, Gottingen, Germany) microscopically examined the stained direct smears. The smear results were reported as per the guidelines published by RNTCP [13]. To measure the inter-observer variability, both technicians were blinded to each other’s microscopic results when examining the direct AFB smears. To check the inter-observer variability, all the positive slides (*n* = 91) and 10% of randomly picked negative slides (*n* = 60) were examined by the study supervisor.

#### 2.2.3. NALC-NaOH DDM

The second aliquot of homogenized sputum (2 mL) was subjected to NALC-NaOH DDM as described by Kent & Kubica with minor changes (final NaOH concentration of 1%) [14]. After the NALC-NaOH DDM procedure, 0.2 mL of the resulting pellet was cultured on Löwenstein–Jensen (LJ) media (two tubes, namely; one LJ tube containing sodium pyruvate and the other tube with glycerol). Each cultured LJ tubes was monitored weekly once during the eight weeks of aerobic incubation (at 37 °C).

#### 2.2.4. Pancreatin-CPC DDM

The homogenized sputum sample (2 mL) was transferred to Falcon tube (14 mL capacity) (BD Biosciences, San Jose, USA), added with an equal quantity of 4% pancreatin (Himedia, Mumbai, India) −4% CPC (Himedia, Mumbai, India) working solution. This working solution was prepared by mixing an equal quantity of 4% pancreatin and 4% CPC stock solution, which was prepared in distilled water (DW)]. Then this reaction tube was subjected to vortexing (1 min) followed by 30 min incubation (at 37 °C). After incubation, DW was added and volume brought until the brim of tube followed by thorough mixing by vortexing and inversion. Later, this tube was centrifuged (3300× *g*) for 20 min and sediment was separated after decanting the supernatant. The sediment was re-suspended with 200 µL of DW and the resuspended pellet (0.2 mL) was cultured on two LJ culture tubes (one media with sodium pyruvate and the other with glycerol). These LJ slants were incubated (aerobically) at 37 °C and checked for growth once every seven days for up to eight weeks.

### 2.3. Identification of Mycobacteria Cultures

The growth of mycobacteria was presumptively identified as per guidelines published by WHO [15]. Quality control of LJ slants was achieved using control strains [*M. tuberculosis* (ATCC H37Rv) and *M. kansasii* (ATCC 12478)]. Polymerase chain reaction (PCR) (primer sequence specific for ‘IS6110’) was used to confirm the MTB isolates. The primer sequence used includes; T4- 5′-CCT GCG AGC GTA GGC GTC GG 3′ and T5-5′ CTC GTC CAG CGC CGC TTC GG 3′ with an expected band size of 123 bps [16].

### 2.4. Statistical Analysis

Diagnostic accuracies of NALC-NaOH DDM (reference methods) were calculated and compared against pancreatin-CPC DDM. The significance of differences in the sensitivities of NALC-NaOH DDM and pancreatin-CPC DDM were assessed using McNemar’s test. The significance of differences in the rate of detection of mycobacteria was analyzed using Fisher’s exact test carried out by GraphPad Prism 6 (Graph-Pad Software, Inc., La Jolla, CA, USA).

## 3. Results

**Figure 1:**
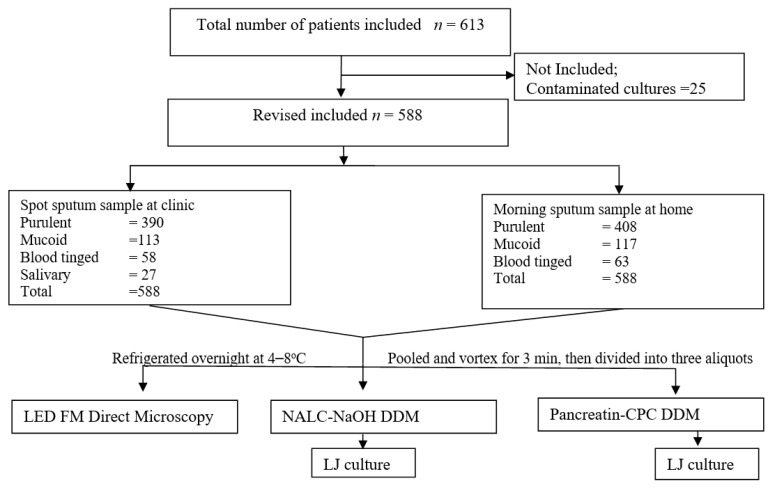
Flow chart showing study profile.

Of 613 sputum specimens processed, direct AFB smear could detect a total of 14.85% (91/613) smear-positive cases. However, LJ detected the presence of MTB and NTM in 22.83% (140/613) and 1.63% (10/613) of the specimens, respectively when treated with NALC-NaOH DDM (reference method). In contrast, sputum samples when digested and decontaminated using pancreatin-CPC DDM, the culture positivity of MTB and NTM were 25.77% (158/613) and that of NTM 2.61% (16/613), respectively. Figure 1 depicts the study profile. Table 1 shows overall results (direct microscopy and culture) obtained after pretreatment with NALC-NaOH and pancreatin-CPC DDMs. Further, 2.28% (14/613) and 3.1% (19/613) of specimens were contaminated in LJ culture after pretreatment with NALC-NaOH DDM and pancreatin-CPC DDM, respectively. After excluding the 4.07 % (25/613) of contaminated culture (obtained after pretreatment with all the methods studied), the culture positivity was found to be 23.64% (139/588) and 1.7% (10/588) for MTB and NTM infections, respectively (after pretreatment with NALC-NaOH DDM). Similarly, after excluding 4.07% (25/613) contaminated culture results, 26.87 % (158/588) and 2.72% (16/588) patients were confirmed with MTB and NTM infections (in culture), respectively (after pretreatment with pancreatin-CPC DDM).

Table 2 shows the comparison of isolation rates of MTB and NTM using LJ culture after pretreatment with alkali and CPC-based DDMs as demonstrated by LED-FM smear scores and quality of sputum. Of the total smear-positive sputum samples (*n* = 91), 60.4% (90/149) of mycobacterial isolates were grown in LJ culture after treatment with alkali-based NALC-NaOH DDM (Table 2). Table 2 illustrates that LJ culture (LJ, *n* = 174, after treatment with pancreatin-CPC DDM) detected twenty-five additional mycobacterial isolates in comparison with alkali-based DDM (LJ, *n* = 149) (Fisher’s exact test, *p* < 0.05). Table 3 shows the culture results yielded after digestion and decontamination with NALC-NaOH and pancreatin-CPC DDM as shown by LED-FM direct microscopy. Interestingly, a direct relationship between direct LED FM smear scores and culture yield was noted. Of the total MTB isolates yielded after treatment with alkali-based (*n* = 149), and CPC (*n* = 174) based DDMs, 29.53% (44/149) and 27% (47/174) sputum samples were having 3+ and 2+ smear scores. These samples also showed heavy growth (3+ and 2+ growth) in LJ culture (Table 3). In contrast, the majority of the sputum samples with low bacillary load (such as sputum samples with 1+ and scanty smear scores) showed comparatively fewer numbers of mycobacterial colonies in culture.

Table 4 shows the growth detection time of LJ culture for recovery of MTB and NTM after treatment with alkali and CPC-based DDMs as indicated by direct microscopy. Table 4 depicts that most of the sputum samples possessing 3+ and 2+ smear grades showed growth of tubercle bacilli within 2–4 weeks (LJ, *n* = 40), irrespective of the type of DDMs used. Alternatively, delayed growth of MTB was noted in samples with 1+ or scanty smear grades. Nevertheless, the differences between the meantime to detection of MTB (on LJ media) for CPC-based DDM was 36.23 days (SD 10.06), which was relatively less when compared to alkali-based DDM (38.3 days; SD 9.72). Likewise, LJ culture showed growth of NTM with a mean detection time of 20.4 (SD 3.29) and 15.94 days (SD 3.5) for samples treated with alkali-based DDM and CPC-based DDM, respectively. Table 5 demonstrates sensitivity and specificity of 99.32% and 94% for LJ culture (when specimens treated with pancreatin-CPC DDM) in comparison with alkali-based DDM (McNemar’s test, *p* < 0.0001) to isolate mycobacterial species (Table 5).

## 4. Discussion

Tuberculosis remains the foremost cause of death across the globe, particularly in Southeast Asian countries. Microbiological detection of TB is essential to stop the progression of TB [1]. Diagnosis of TB can be made by direct microscopic examination, culture, histological and radiological examinations [2]. However, culture is regarded as the gold standard to diagnose active TB. Further, effective isolation of MTB in culture depends on the type of DDMs used [2]. This study compared the functioning of NALC-NaOH DDM with newly developed pancreatin-CPC DDM. In the present study, patients who received ATT in the last six months were excluded as ATT may influence the viability of MTB in culture irrespective of the duration of treatment. The other characteristic of the current study design was the mixing of spot sputum samples with early morning sputum samples to enhance the quality of sputum; indeed, the majority of the samples collected at the hospital with a mucoid or salivary consistency, and when this sputum was pooled with morning sample, a purulent consistency was obtained.

In this study, culture detected the presence of MTB in 22.84% (140/613) of the specimens when treated with NALC-NaOH DDM in comparison with direct AFB smear 14.84% (91/613). These results were consistent with earlier works as well, in which they investigated various rapid methods for the detection of MTB in parallel with conventional LJ culture [2]. However, a higher detection rate of MTB was noted when specimens were treated with pancreatin-CPC DDM (detection rate of MTB was 25.77%) signifying the impending nature of pancreatin-CPC DDM for the effective isolation of MTB than NALC-NaOH DDM. Further, of the total samples processed using NALC-NaOH DDM, NTM growth was observed in 1.63% of cases, which was comparable with earlier works published from this geographical location [17,18].

The isolation rate of NTM was considerably increased when sputum specimens were treated with pancreatin-CPC DDM (LJ, 2.61%) suggesting the potential of pancreatin-CPC DDM for the effective isolation of NTM as well. In the current study, the rate of contamination was found to be low for sputum samples treated with NALC-NaOH (1.8%) and pancreatin-CPC DDM (3.09%) and the differences in the contamination rate were insignificant. This minimum rate of contamination of the NALC-NaOH decontaminated samples may be because of a low concentration of NaOH. Nevertheless, the relatively low contamination rate of pancreatin-CPC DDM may be endorsed due to the bacteriostatic action of CPC, which is a quaternary ammonium compound that disrupts bacterial cell membranes (especially the commensal bacteria in the upper respiratory tract) and subsequently resulting in the death of bacteria.

LED FM continues to be the primary diagnostic tool for peripheral TB laboratories and peripheral health centers with limited resources [13]. In this study, LED FM documented sensitivity and specificity of 60.4% and 99.77%, respectively. This microscopic technique revealed a diagnostic accuracy of 60.4% (sensitivity) and 99.77% (specificity), respectively. It was consistent with previous studies wherein the diagnostic accuracy for the LED FM ranged between 65–85% and 80–90%, respectively [19,20]. Further, direct LED microscopy detected one smear-positive but culture-negative case after treatment with the NALC-NaOH method. Conversely, the same cases were detected by LJ culture after treatment with pancreatin-CPC DDM. This culture-negative but smear-positive (false positive) results perhaps due to the death of tubercle bacilli caused by the alkaline toxicity that must have been induced during the NALC-NaOH DDM procedure [2]. However, culture possesses many advantages over LED FM, indeed; LJ culture detected additional cases of mycobacteria in 10% (59/588) and 14.12% (83/588) of sputum specimens when treated with NALC-NaOH and pancreatin-CPC DDMs, respectively. This increased detection rate of culture may be imputed to the diagnostic accuracy of culture, which is used to isolate tubercle bacilli particularly in samples with low bacillary load [21]. Culture exhibited an early growth of tubercle bacilli in most of the samples (LJ, *n* = 40) that possess higher smear grades regardless of the pre-treatment method used (Table 4). However, the meantime to detection of bacillary growth was increased (6 weeks or more) in specimens having low smear grades (1+ and scanty). This can be explained based on smear scores; more specifically, smear scores depend on the number of bacilli present in the specimens. Therefore, specimens with higher smear scores, show reduced growth detection time. 

Recently, various DDMs have been investigated and most of these DDMs use a chemical agent for digestion and the other as a disinfectant [2,4,5,6,7,8,9,10]. The most commonly used NALC-NaOH digestion and decontamination technique uses 0.5% NALC as a mucolytic agent and NaOH (final concentration of 1%) as a decontaminating agent [14]. In the current study, culture positivity was relatively high exclusively for smear-negative samples (=59) after treatment with alkali-based DDM. This increased isolation rate of mycobacteria could be attributed to the improved sensitivity of culture, which is higher than that of microscopy. Further, culture detected growth of MTB in most of the samples (when treated with NALC-NaOH DDM) and it was noted that all these strains were found to be grown in LJ culture after treatment with pancreatin-CPC DDM as well. This is probably due to the chemical properties of the various samples and their response to various pre-treatment procedures. The other limitations of NALC-NaOH DDM include (a) the duration of pretreatment of the specimen with alkali is limited, indeed; as the duration of alkaline treatment is increased, that may cause toxic effect on few strains MTB. Therefore, the duration of alkaline exposure is vital and limited to 20 min. (b) need to adjust the pH of the NALC-NaOH DDM during the pre-treatment process. 

In pancreatin-CPC DDM, pancreatin (4%) and CPC (4%) function as digestive, and decontaminating agents, respectively. The particular concentration of 4% pancreatin−4% CPC used in this study was determined after conducting a pilot study using varying concentrations of pancreatin. It was noted that overnight treatment of clinical samples with 1% CPC could decontaminate the samples effectively [2]. However, the current study used a higher concentration of CPC (4%) to minimize the duration of pretreatment (such as 30 min). Considering these points in mind, we tested varying concentrations of pancreatin 0.5%, 1%, 2%, 3%, 4% and 5% in combination with 4% CPC against AFB smear-positive sputum samples (*n* = 10). This reaction mixture was incubated at 37 °C for 30 min. The results of the pilot study suggested that 4%concentration of both pancreatin and CPC might be effective for isolation of MTB. In this study, culture detected a significant number of MTB isolates when smear-negative samples were treated with pancreatin-CPC DDM (LJ culture, *n* = 83) than those specimens when exposed to NALC-NaOH DDM (LJ culture, *n* = 59). The differences between these were found to be significant (Fisher’s exact test, *p* < 0.05). These increased false-negative results can be endorsed to the lytic property of pancreatin, which contains a mixture of lytic enzymes that could disintegrate the sputum samples to discharge the bacilli embedded in the mucus clumps and macrophages. It was also noted that the growth detection time was relatively reduced when specimens treated with pancreatin-CPC DDM as compared to those specimens that were treated with NALC-NaOH DDM (Table 4). This is possible may be due to the nontoxic action of CPC on the viability of mycobacterial species. This further suggests that pancreatin-CPC DDM may be used as an alternative method for NALC-NaOH DDM. Pancreatin-CPC DDM also possesses several advantages over NALC-NaOH DDM. This includes the stability of pancreatin-CPC solution (shelf life of one week once constituted), easy preparation, autoclaving of the pancreatin-CPC solution is not required as CPC itself a self-decontaminating solution, washing with distilled water is not required before the inoculation on LJ media, and thereby spares the time of technologists. The approximate cost for pancreatin-CPC and NALC-NaOH DDM was found to be ~5 ₹ and ~10 ₹, respectively. However, the pancreatin-CPC DDM also possesses few disadvantages like the possibility of crystal formation while the working solution is kept at a low temperature (<22 °C) [6]. However, this problem can be resolved when these solutions are kept at a temperature >25 °C. Another limitation of CPC-treated specimens includes a relatively low isolation rate of MTB when the CPC-treated specimens are cultured in BBL^TM^ MGIT^TM^ tubes (as they may interfere with fluorescent signals of BBL MGIT) [22]. However, this negative effect of CPC-treated specimens can be minimized, when all the sediments obtained after treatment with pancreatin-CPC DMM may be washed with phosphate buffer before inoculation into BBL MGIT tubes.

## 5. Conclusions

In summary, LJ culture showed an incremental yield in the recovery of mycobacterial isolates mainly from smear-negative sputa that were treated with pancreatin-CPC DDM than NALC-NaOH DDM treated sputa, suggesting its promising use in clinical TB laboratories. 

## Figures and Tables

**Table 1:** Overall results (direct microscopy and culture results) were obtained after digestion and decontamination with NALC-NaOH (*n*-Acetyl-L-Cysteine-Sodium hydroxide) and Pancreatin-CPC (Cetyl Pyridinium Chloride) DDMs.

Culture Results	Direct LED FM Smear Positive Cases		Total Samples Processed	
	NALC-NaOH DDM	Pancreatin-CPC DDM	NALC-NaOH DDM	Pancreatin-CPC DDM
	LJ Culture (*n* = 91) %	LJ Culture (*n* = 91) %	LJ Culture (*n* = 613) %	LJ Culture (*n* = 613) %
^a^ MTB	85 (93.4)	86 (94.5)	140( 22.83)	158 (25.77)
^b^ NTM	5 (5.49)	5 (5.49)	10 (1.63)	16 (2.61)
No growth	1(1.09)	−	457 (74.55)	428 (69.82)
Contaminated cultures	−	−	14 (2.28)	19 (3.1)
Bacterial	−	−	9 (1.47)	13 (2.12)
Fungal	−	−	−	2 (0.33)
Liquefied LJ media	−	−	5 (0.82)	4 (0.65)

^a^ Mycobacterium tuberculosis; ^b^ Non tuberculosis mycobacteria; LJ, Löwenstein-Jensen.

**Table 2:** Comparison of isolation rates of MTB and NTM using LJ culture (after pretreatment with NALC-NaOH DDM and Pancreatin-CPC DDM) as demonstrated by LED-FM smear scores and quality of sputum.

NALC-NaOH Digestion and Decontamination Methods													Pancreatin-CPC Digestion and Decontamination Methods										
LJ Culture													LJ Culture										
Quality of sputum	* LED FM smear score												* LED FM smear score										
	3+ (*n* = 34)		2+ (*n* = 18 )		1+ (*n* = 25)		Scanty (*n* = 14)				Negative (N497)		3+ (*n* = 34)		2+ (*n* = 18 )		1+ (*n* = 25)		Scanty (*n* = 14)			Negative (*n* = 497)	
	MTB	NTM	MTB	NTM	MTB	NTM	MTB	NTM	NG	MTB	NTM	NG	MTB	NTM	MTB	NTM	MTB	NTM	MTB	NTM	MTB	NTM	NG
Purulent(*n* = 408)	22	−	8	1	9	1	5	2	−	52	1	307	22	−	8	1	9	1	5	2	59	3	298
Mucoid(*n* = 117)	9	−	4	−	9	−	4	−	−	2	2	87	9	−	4	−	9		4		8	2	81
Blood tinged(63)	3	−	5	−	6	−	1	1	1	−	2	44	3	−	5	−	6		2	1	5	6	35
Subtotal	34	−	17	1	24	1	10	3	1	54	5	438	34	−	17	1	24	1	11	3	72	11	414
Total number of culture isolates	**149**												**174**										

* as per Revised National Tuberculosis Control Program; NG—No growth.

**Table 3:** Culture results yielded after digestion and decontamination with NALC-NaOH and Pancreatin CPC DDM as shown by LED-FM direct microscopy.

Direct Smear Scores (LED FM)	Culture on LJ Media																	
	NALC-NaOH DDM									Pancreatin-CPC DDM								
	^a^ 3+ (*n* = 45 *)		^b^ 2+ (*n* = 28 *)		^c^ 1+ (*n* = 31 *)		^d^ Scanty (*n* = 45 *)		NG (*n* = 439)	^a^ 3+ (*n* = 63 *)		^b^ 2+ (*n* = 31 *)		^c^ 1+ (*n* = 34 *)		^d^ Scanty (*n* = 46 *)		NG (*n* = 414)
	MTB	NTM	MTB	NTM	MTB	NTM	MTB	NTM		MTB	NTM	MTB	NTM	MTB	NTM	MTB	NTM	
3+ (*n* = 34)	19	−	12	−	2	−	1	−	−	24	−	7	−	3	−	−	−	−
2+ (*n* = 18)	7	1	5	−	4	−	1	−	−	11	1	4	−	2	−	−	−	−
1+ (*n* = 25)	6	−	1	−	7	1	10	−	−	8	−	5	−	7	1	4	−	−
Scanty (*n* = 14)	2	1	1	2	3	−	4	−	1	2	1	5	1	1	1	3	−	−
Negative (*n* = 497)	7	2	5	2	13	1	29	−	438	9	7	6	3	18	1	39	−	414
Total number of culture isolates				149										174				

^a^ 3+—more than 200 colonies; ^b^ 2+—100 to 200 colonies; ^c^ 1+—20 to 99 colonies; ^d^ Scanty—less than 20 colonies; NG—No growth; * indicates presence of non-tuberculous mycobacteria.

**Table 4:** Growth detection time of LJ culture for recovery of *Mycobacterium tuberculosis* (MTB) and non-tuberculous Mycobacteria (NTM) after digestion and decontamination with NALC-NaOH and pancreatin-CPC DDM as indicated by LED-FM smear scores.

Growth Detection Time (Weeks)	LED-FM Direct Smear Scores and Culture Results									
	NALC-NaOH DDM					Pancreatin-CPC DDM				
	LJ Culture					LJ Culture				
	3+ *n* = 34	2+ *n* = 18	1+ *n* = 25	Scanty *n* = 13	Negative *n* = 59	3+ *n* = 34	2+ *n* = 18	1+ *n* = 25	Scanty *n* = 14	Negative *n* = 83
MTB										
≤1	−	−	−	−	−					
2	11	7	1	−	1	13	10	2	−	4
3	12	5	1	−	6	9	2	−	1	11
4	3	1	4	2	7	5	3	3	−	16
5	5	3	6	1	2	3	−	7	3	10
6	1	1	5	2	9	2	1	9	5	5
7	2	−	5	3	12	2	1	2	−	19
8	−	−	2	2	17	−	−	1	2	7
NTM										
≤1	−	−	−	1	−	−	1	1	−	3
2	−	−	−	−	1	−	−	−	1	2
3	−	1	1	−	1	−	−	−	2	5
4	−	−	−	2	3	−	−	−	−	1
5	−	−	−	−	−	−	−	−	−	
6	−	−	−	−	−	−	−	−	−	
7	−	−	−	−		−	−	−	−	
8	−	−	−	−		−	−	−	−	

**Table 5:** Diagnostic accuracy of direct microscopy and culture for detection of *Mycobacterium tuberculosis* (MTB) and non-tuberculous Mycobacteria (NTM) after treatment with NALC-NaOH and pancreatin-CPC DDMs.

Methods		Culture		Sensitivity (%)	95% C.I.	Specificity (%)	95% C.I.	PPV (%)	95% C.I.	NPV (%)	95% C.I.
		Positive	Negative								
* Direct LED-FM smear	Positive	90	1	60.4	52.04–68.21	99.77	98.53–99.98	98.9	93.17–99.94	88.3	84.88–90.78
	Negative	59	438								
Pancreatin-CPC DDM											
* LJ culture	Positive	148	26	99.32	95.75–99.96	94.07	91.33–96	85.05	78.68–89.83	99.76	98.45–99.98
	Negative	1	413								

* Number of specimens positive for *Mycobacterium tuberculosis* and non-tuberculous mycobacteria (*n* = 149) and negative for mycobacteria (*n* = 429) after treatment with NALC-NaOH DDM for calculating diagnostic accuracy of LJ culture.

## Data Availability

All relevant data are provided in the manuscript.
